# Glycogen and lactate metabolism in mouse fetal Sertoli cells sustain the germ line

**DOI:** 10.1016/j.celrep.2026.117069

**Published:** 2026-03-10

**Authors:** Martín A. Estermann, Joseph Sheheen, Sara A. Grimm, Boris Tezak, Yu-Ying Chen, Tsuyoshi Morita, Humphrey H.-C. Yao, Blanche Capel

**Affiliations:** 1 Reproductive Developmental Biology Group, National Institute of Environmental Health Sciences, Research Triangle Park, NC 27709, USA; 2 Department of Cell Biology, Duke University Medical Center, Durham, NC 27701, USA; 3 Integrative Bioinformatics, National Institute of Environmental Health Sciences, Research Triangle Park, NC 27709, USA; 4 Biology Department, Wesleyan University, Middletown, CT 06459, USA; 5 Oral and Maxillofacial Anatomy, Tokushima University Graduate School, Tokushima 770-8504, Japan; 6 These authors contributed equally; 7 These authors contributed equally; 8 Lead contact

## Abstract

Metabolites are key regulators of cell fate decisions, chromatin remodeling, and lineage commitment. While genetic pathways governing testis differentiation are well studied, the role of metabolism remains poorly understood. In this study, we investigate the transient, male-specific accumulation of glycogen in supporting cells of the fetal testis in mice, between embryonic days 11.5 and 13.5. Blocking glycogen metabolism/accumulation *in vivo* and *in vitro* is dispensable for Sertoli cell differentiation. However, its disruption leads to reduced lactate production and reduced germ cell number in the testis. Inhibiting lactate transport reveals a critical metabolic coupling between Sertoli and germ cells during early testis development. Surprisingly, external lactate or glucose supplementation fails to rescue the germ cell phenotype. These findings suggest that glycogen accumulation supports a critical developmental window in which both Sertoli and germ cells are metabolically constrained and unable to rely on external carbon sources.

## In brief

Estermann and Sheheen et al. identified a metabolic coupling between fetal Sertoli and germ cells in mice, driven by glycogen breakdown and lactate transport through the MCT4/MCT1 shuttle. This interaction is essential to support fetal germ cell development.

## Graphical Abstract

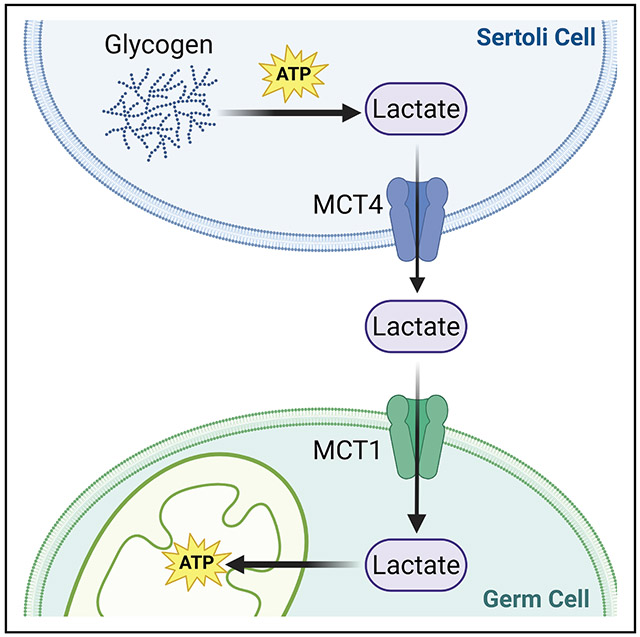

## INTRODUCTION

Increasing evidence across developmental systems indicates that metabolism is far more than a background process supporting biosynthesis and energy production. It is an active regulator of cell fate decisions, chromatin remodeling, and lineage commitment.^1-6^ In this context, the concept of metabolic coupling, the coordinated exchange of metabolites between specialized cells, has emerged as a key mechanism by which cell populations communicate, synchronize their developmental trajectories, and compartmentalize their functions. This mechanism, often exploited by cancer cells,^7-9^ plays a vital role in the normal function of many organs, including in the brain, kidney, muscle, and adult testis.^10-13^ In the adult testis, Sertoli cells provide metabolic and functional support to germ cells to sustain sperm production.^14-18^ However, when this metabolic coupling is established during testis development has not been explored.

Fetal testicular differentiation involves the transformation of the bipotential gonadal primordium into a testis, a process orchestrated by the supporting cell lineage.^19^ Supporting cells are the first to commit to a testicular (Sertoli) fate around embryonic day 11.5 (E11.5) in mice.^20-22^ Once specified, Sertoli cells coordinate the differentiation of the rest of the gonadal cell types, including the germline.^22-24^ While the genetic regulators driving these processes have been well characterized, much less is known about how metabolic cues influence sexual fate decisions.

A puzzling early metabolic difference between XY and XX gonadal supporting cells at the onset of sex determination is the accumulation of glycogen deposits in male, but not female, supporting cells.^25,26^ Despite this observation, the exact role of this sex-specific metabolic difference has not been clearly identified. To explore this metabolic dimorphism, we perturbed glycogen synthesis and breakdown pathways. We show that while glycogen metabolism is not required for Sertoli cell differentiation, it is required for germ cells to initially populate the fetal testis cords as they form. Disruption of the lactate shuttle produced a similar phenotype. Our results suggest that Sertoli cells break down glycogen to produce lactate, which is then transported to germ cells via the lactate shuttle, supporting their development. These findings establish glycogen and lactate metabolism as critical determinants of germ cell development, highlighting a previously unrecognized metabolic coupling between fetal Sertoli and germ cells, with potential implications for male fertility.

## RESULTS

### Sertoli cells accumulate glycogen within a specific developmental window

**Figure 1.**
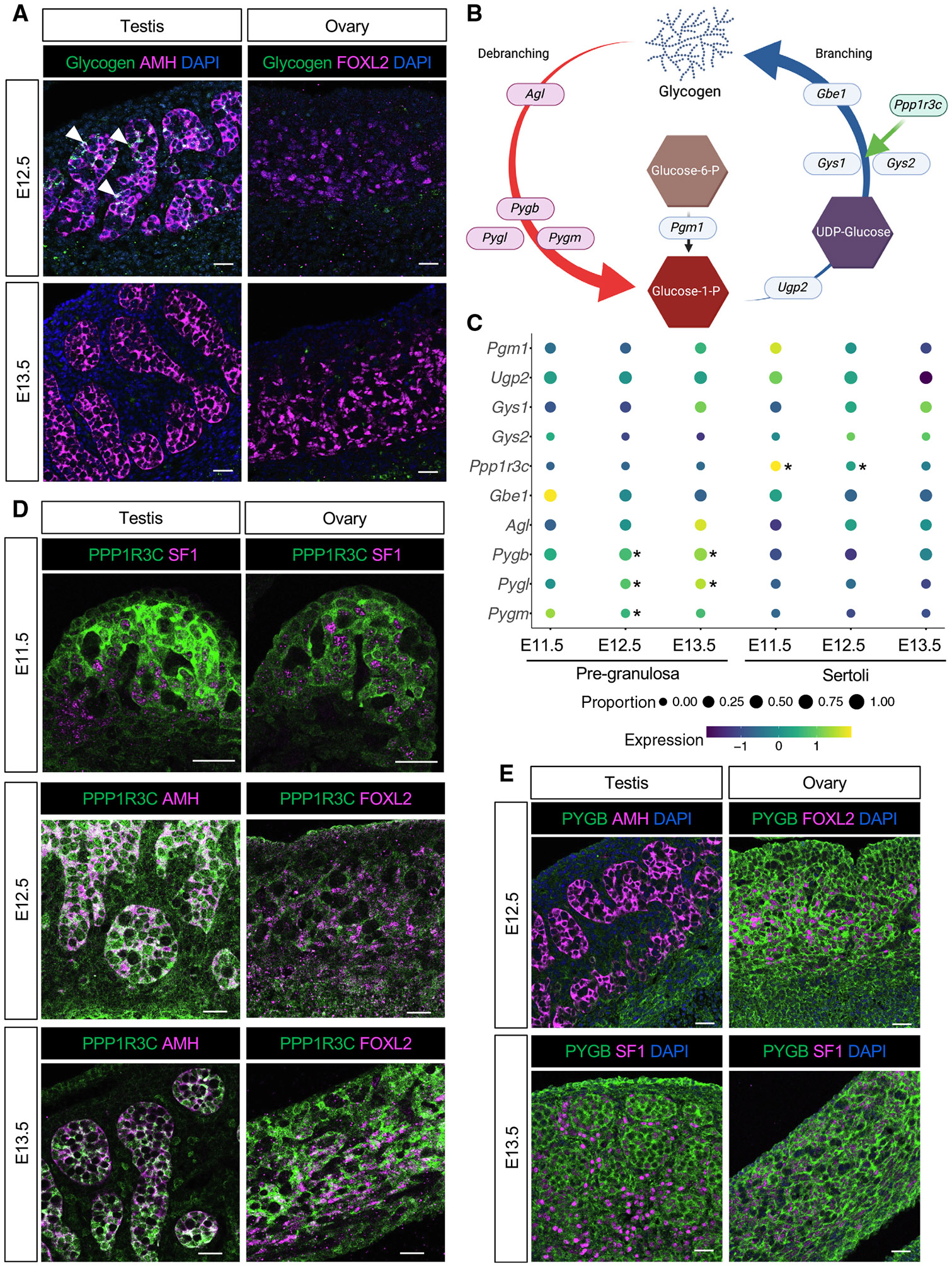
Differential glycogen metabolism results in Sertoli cell-specific accumulation (A) Immunofluorescence for glycogen (green) and male (AMH) or female (FOXL2) supporting cell markers (magenta) in E12.5 and E13.5 gonads. White arrowheads indicate glycogen deposition in Sertoli cells at E12.5. Samples were counterstained with DAPI (blue). Scale bars: 50 μm. (B) Schematic representation of the glycogen synthesis (right) and glycogen degradation (left) pathways (created with BioRender.com). (C) Dot plot representation of the expression of glycogen-pathway-related genes in male (right) and female (left) supporting cells at different developmental timepoints. *Adjusted *p* value (adj*p*) < 0.05, Wilcoxon rank sum test. (D) Immunofluorescence for PPP1R3C (green) and supporting cell marker SF1, AMH, or FOXL2 (magenta) in transverse (E11.5) or longitudinal (E12.5 and E13.5) gonadal sections. Scale bars: 50 μm. (E) Immunofluorescence for PYGB (green) and supporting cell marker SF1, AMH, or FOXL2 (magenta) in E12.5 and E13.5 gonads. Samples were counterstained with DAPI (blue). Scale bars: 50 μm.

To characterize the glycogen deposition pattern, immunofluorescence was performed using a glycogen-specific antibody^27^ on gonadal paraffin sections from sexually differentiating male and female gonads. At E11.5 (ts 20) (Figures S1A and S1B), despite the presence of SRY+ and SOX9+ Sertoli cells, glycogen was not detected using this antibody. As a positive control, the notochord displayed strong glycogen immunoreactivity at E11.5 (Figure S1C). By E12.5, glycogen was detected in Sertoli cells in the testicular cords, colocalizing with a known Sertoli cell marker, AMH (Figure 1A). However, it was absent in FOXL2+ ovarian supporting (pre-granulosa) cells (Figure 1A). Interestingly, by E13.5, glycogen was no longer detectable in the testis (Figure 1A). This suggests a tightly regulated developmental window in which glycogen accumulates in Sertoli cells between E11.5 and E12.5, soon after their differentiation initiates, followed by its breakdown and/or consumption between E12.5 and E13.5.

### Sex-specific glycogen synthesis and degradation result in glycogen accumulation in Sertoli cells

To understand the molecular mechanism underlying glycogen accumulation and subsequent breakdown, we interrogated a mouse gonadal single-nucleus RNA sequencing (RNA-seq) dataset^28^ for the expression of enzymes involved in glycogen synthesis and breakdown in supporting cells across developmental stages (Figures 1B and 1C). No significant differences were observed in the expression of glycogen synthesis enzymes (*Pgm1*, *Ugp2*, *Gys1*, *Gys2,* and *Gbe1*) (Figure 1C). However, Sertoli cells in the embryonic testis expressed significantly higher levels of *Ppp1r3c* at E11.5 and E12.5 than the pre-granulosa cells of the embryonic ovary (Figure 1C; Table S1). *Ppp1r3c*, also known as protein targeting to glycogen (PTG), regulates the phosphorylation (activation) of glycogen synthase (GYS1 and GYS2), therefore promoting glycogen formation. At E11.5 and E12.5, Sertoli cells showed higher PPP1R3C protein expression than pre-granulosa cells, while expression levels were comparable at E13.5 (Figures 1D and S1D). This temporal difference in expression aligns with the pattern of glycogen accumulation observed in the testis (Figure 1A).

In contrast, pre-granulosa cells exhibited significantly higher levels of enzymes that degrade glycogen, *Pygl*, *Pygb,* and *Pygm* at E12.5 and *Pygl* and *Pygb* at E13.5 (Figure 1C; Table S1). PYBG, the most abundant isoform (Figure 1C; Table S1), was expressed at similar protein levels in XX and XY gonads at E11.5 and E13.5 (Figures S1D and S1E). However, testes exhibited lower expression of the PYGB protein at E12.5 (Figure 1E). These data suggest that glycogen accumulates in Sertoli cells at E12.5 as a result of differential synthesis (mediated by PPP1R3C) and reduced glycogen breakdown (mediated by PYG enzymes).

### Glycogen is not required for fate determination of Sertoli cells

**Figure 2.**
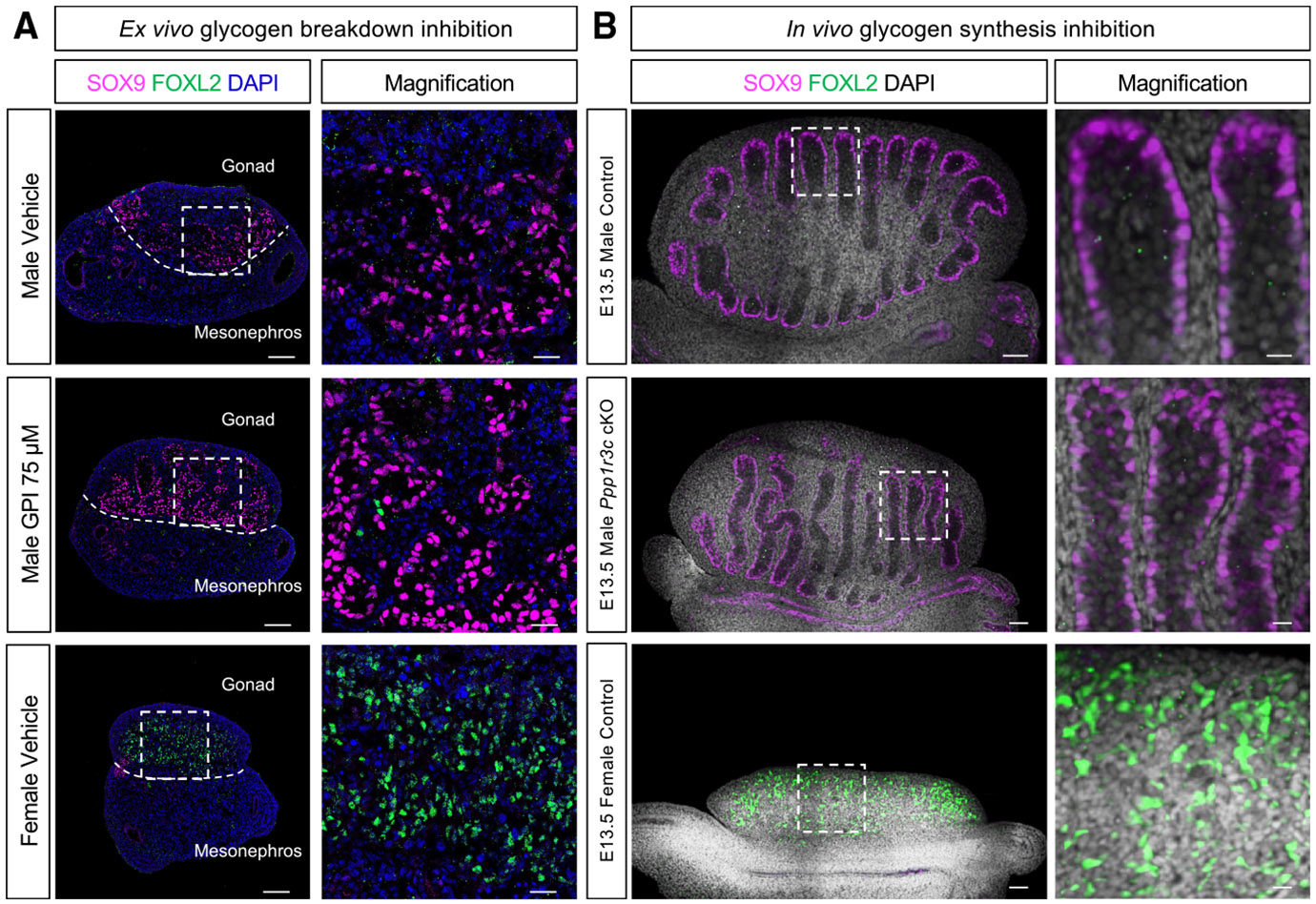
Glycogen is not required for Sertoli cell differentiation (A) Immunofluorescence for the Sertoli cell marker SOX9 (magenta) and pre-granulosa cell marker FOXL2 (green) in E12.5 cultured gonads treated with 75 μM GPI or vehicle solution for 48 h. Samples were counterstained with DAPI (blue). Scale bars: 100 μm. The dashed white line delineates the gonadal region. The white dashed box indicates the magnified area. Scale bars: 25 μm. (B) Immunofluorescence for the Sertoli cell marker SOX9 (magenta) and pre-granulosa cell marker FOXL2 (green) in E13.5 male and female control and male *Ppp1r3c* cKO gonads. Samples were counterstained with DAPI (gray). Scale bars: 50 μm. The white dashed box indicates the magnified area. Scale bars: 12.5 μm.

To investigate the role of glycogen breakdown during gonadal differentiation, we performed *ex vivo* gonadal cultures using the glycogen phosphorylase inhibitor (GPI) CP 316819, which inhibits all 3 glycogen phosphorylase enzymes (PYGB, PYGL, and PYGM). Taking advantage of the gonads being paired organs, we incubated one E12.5 gonad from a single embryo with the GPI inhibitor and the contralateral gonad with a vehicle control (DMSO) for 48 h. To determine the optimal concentration and assess potential toxicity, we cultured gonadal pairs with 75, 100, and 150 μM of GPI or vehicle solutions and evaluated apoptosis via cleaved caspase-3 (CC3) staining and proliferation by staining for Ki-67 (MKI67) (Figure S2A). We selected 75 μM of GPI for further experiments, as higher concentrations increased gonadal apoptosis (Figure S2A). We first examined whether glycogen breakdown was required for Sertoli cell differentiation. Although gonads grew more slowly *ex vivo* and did not retain perfect morphology, male gonads cultured with 75 μM of GPI exhibited normal Sertoli cell differentiation markers, evidenced by the expression of SOX9 and the absence of FOXL2, a marker of pre-granulosa cells (female vehicle control), which would be indicative of sex reversal (Figure 2A). These findings suggest that glycogen breakdown is not required for Sertoli cell differentiation or fate maintenance.

To evaluate the role of glycogen synthesis in Sertoli cells, we obtained a *Ppp1r3c* conditional mouse line^29^ and generated *Ppp1r3c* conditional knockout (cKO) mice using *Nr5a1*-Cre, a Cre expressed in Sertoli cell progenitors. To confirm that deletion of *Ppp1r3c* leads to the abrogation of glycogen accumulation in Sertoli cells, we performed periodic acid-Schiff (PAS) staining, which detects the presence of polysaccharides, including glycogen, on E12.5 control or *Ppp1r3c* cKO gonads (Figure S2B). In control gonads, as expected, PAS+ staining was detected in Sertoli cells (Figure S2B), consistent with our previous results (Figure 1A). In contrast, PAS staining was absent in cKO testes, similar to results in the ovary (Figure S2B), confirming that loss of *Ppp1r3c* blocks glycogen synthesis and its accumulation. Immunofluorescence for Sertoli (SOX9) and pre-granulosa (FOXL2) cell markers indicated that loss of *Ppp1r3c* in Sertoli cells did not affect Sertoli cell differentiation (Figure 2B). These results collectively demonstrate that neither the synthesis nor the degradation of glycogen is required for Sertoli cell fate determination.

### Altered glycogen metabolism in Sertoli cells results in reduced germ cell number

**Figure 3.**
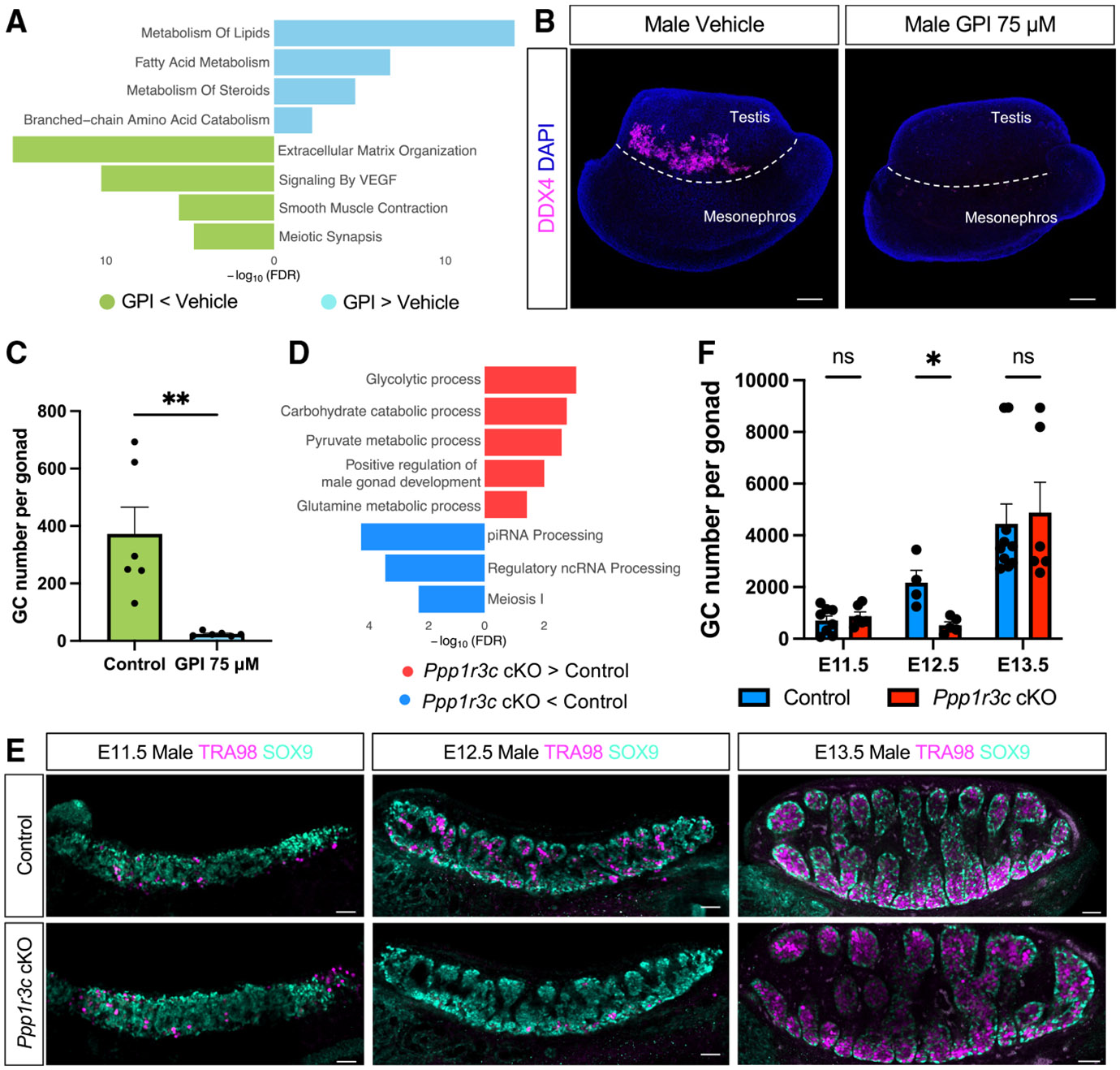
Blocking glycogen breakdown or synthesis reduces germ cell number in XY gonads (A) Reactome pathway analysis of significant up-regulated (cyan) and downregulated (green) genes in the GPI-treated gonads vs. the vehicle control. (B) 3D projection of DDX4 (germ cell, magenta) whole-mount immunostaining in male gonads cultured with 75 μM GPI or vehicle solution. Samples were counterstained with DAPI (blue). The dashed white line delineates the testicular region. Scale bars: 100 μm. (C) Quantification of the total number of germ cells in male gonads cultured with 75 μM GPI or vehicle solution. Bars represent the mean ± SEM; *n* = 6. Two-tailed *t* test. **p < 0.01. (D) Gene Ontology (BP) analysis of significant upregulated (red) and downregulated (blue) genes in E12.5 *Ppp1r3c* cKO testis vs. control. (E) Immunofluorescence for the Sertoli cell marker SOX9 (green) and germ cells using the TRA98 antibody (magenta) in E11.5, E12.5, and E13.5 male control and *Ppp1r3c* cKO gonads. Scale bars: 50 μm. (F) Quantification of the total number of germ cells in male control and *Ppp1r3c* cKO testes. Bars represent the mean ± SEM; *n* ≥ 4. Multiple two-tailed *t* test. ns adj*p* > 0.05 and *adj*p* < 0.05.

To further investigate the role of glycogen breakdown in gonadal differentiation, we performed bulk RNA-seq on paired E12.5 testes cultured with 75 μM of GPI or vehicle solution for 48 h. A total of 5 testicular pairs were sequenced, and principal-component analysis (PCA) revealed a clear separation by treatment (Figure S3A). Paired differential expression analysis identified 3,202 differentially expressed genes (DEGs), including 1,770 downregulated and 1,432 upregulated in the GPI-treated testes compared to controls (Figure S3B; Table S2). Consistent with our previous results, Sertoli cell key markers were not downregulated, suggesting that Sertoli cell differentiation (*Sox9* and *Dmrt1*) and function (*Amh* and *Inhbb*) were not affected upon glycogen breakdown inhibition (Figure S3B; Table S2). Pathway analysis of the upregulated genes identified lipid metabolism among the most enriched pathways (Figure 3A; Table S2), suggesting a metabolic shift from glucose oxidation to fatty acid metabolism. Pathway analysis of the downregulated genes identified an impairment of genes involved in extracellular matrix organization and in germ cell development (Figure 3A). To determine whether these transcriptional changes in germ cell gene expression reflected changes in germ cell numbers, we performed whole-mount immunofluorescence for the pan-germ cell marker DDX4 (Figure 3B) and quantified the germ cell numbers in control and GPI-treated testes (Figures 3B and 3C). GPI-treated testes exhibited a significant reduction in germ cell number, with an average of 91% fewer germ cells, compared with the control (Figures 3B and 3C), suggesting that glycogen degradation in Sertoli cells is essential for controlling germ cell number of the gonad.

As the GPI treatment targets all gonadal cells, the role of Sertoli-specific glycogen metabolism in gonadal differentiation was evaluated using our mouse cKO model in which *Ppp1r3c* is deleted specifically in Sertoli cells. Bulk RNA-seq in control and *Ppp1r3c* cKO E12.5 testis (Figure S3C) and differential expression analysis identified 111 DEGs, 57 upregulated and 54 downregulated (Figure S3D; Table S3). Notably, *Ppp1r3c* was the top downregulated gene, with a 76.8% reduction, confirming a robust gene knockout (Figure S3D). Consistent with our previous results, Sertoli cell key markers were not significantly modulated, suggesting that Sertoli cell differentiation (*Sox9* and *Dmrt1*) and function (*Amh* and *Inhbb*) were not affected upon inhibition of glycogen deposition (Figure S3D; Table S3). Pathway analysis of the differentially upregulated genes revealed enrichment in carbohydrate metabolism pathways, suggesting either a compensatory metabolic response to glycogen loss or possibly more available glucose in the absence of glycogen reservoirs (Figure 3D; Table S3). Interestingly, and consistent with our inhibitor results, downregulated genes were associated with germ cell functions, including piRNA processing and meiosis (Figure 3D). To identify which gonadal cell population was most affected by Sertoli cell-specific *Ppp1r3c* deletion, we assessed the expression patterns of the 54 downregulated genes from our bulk RNA-seq analysis within our mouse gonadal single-nucleus RNA-seq dataset.^28^ Notably, almost all downregulated genes (50/54) showed enriched expression in germ cell-specific clusters (Figure S3E), indicating that the transcriptional changes observed in our bulk analysis primarily reflect an impact on the germline. To determine whether these transcriptional changes in germ cell gene expression reflected changes in germ cell numbers, we performed whole-mount immunofluorescence using the germ cell-specific antibody TRA98^30^ (Figure 3E) and quantified germ cell numbers in control and *Ppp1r3c* cKO testes (Figure 3F). While germ cell numbers were comparable between control and cKO gonads at E11.5 (Figures 3E and 3F), by E12.5, a significant reduction of germ cell number was observed in *Ppp1r3c* cKO testes (Figures 3E and 3F). However, by E13.5, as glycogen deposition declined in Sertoli cells (Figure 1A), germ cell numbers recovered (Figures 3E and 3F). Taken together, these findings suggest Sertoli cell glycogen deposits are utilized by germ cells to populate the fetal testis during a transient window of development.

### Sertoli and germ cells are metabolically coupled through the lactate shuttle

As their name suggests, supporting cells play a crucial role in nourishing the germline, raising the possibility that Sertoli cells and germ cells are metabolically coupled. Because lactate is a common product of glycogen breakdown^31^ and because meiotic and post-meiotic germ cells in the adult testis rely on lactate provided by Sertoli cells,^14-18^ we investigated whether this system might underlie the effects of glycogen loss on germ cell numbers.

**Figure 4.**
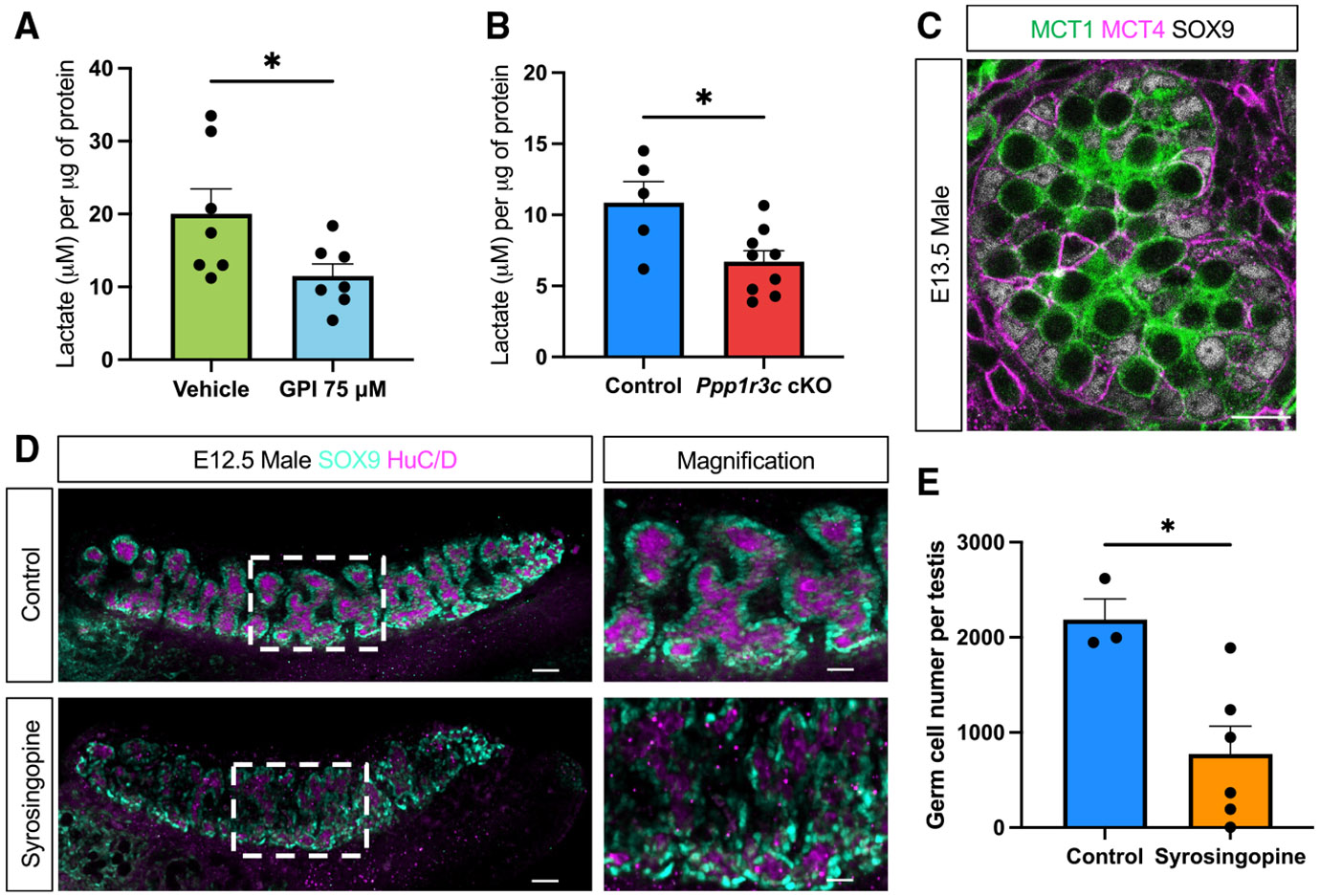
Blocking glycogen breakdown or synthesis reduces germ cell number in XY gonads during sex determination (A) Lactate concentration in testis cultured with 75 μM GPI or vehicle solution. Bars represent the mean ± SEM; *n* = 7. Two-tailed *t* test. *p < 0.05. (B) Lactate concentration in E12.5 *Ppp1r3c* cKO or control testes. Bars represent the mean ± SEM; *n* ≥ 5. Two-tailed *t* test. *p < 0.05. (C) Immunofluorescence for MCT1 (green), MCT4 (magenta), and SOX9 (gray) in E13.5 male gonads. Scale bars: 12 μm. (D) Immunofluorescence for the Sertoli cell marker SOX9 (cyan) and germ cell marker HuC/D (magenta) in E12.5 male control and syrosingopine-treated gonads. Scale bars: 50 μm. The white dashed box indicates the magnified area. Scale bars: 25 μm. (E) Quantification of the total number of germ cells per testis from control and syrosingopine-treated mice. Bars represent the mean ± SEM; *n* ≥ 3. Two-tailed *t* test. *p < 0.05.

As our findings suggest that Sertoli cells break down their stored glycogen to fuel the germline, we hypothesized that glycogen is oxidized into lactate. Indeed, both inhibition of glycogen breakdown using GPI and inhibition of glycogen synthesis in the *Ppp1r3c* cKO model resulted in an approximately 40% reduction in intracellular lactate levels in embryonic testes (Figures 4A and 4B), strongly suggesting that lactate is a significant by-product of glycogen breakdown in early Sertoli cells.

Lactate moves between cells through the exporter MCT4 (*Slc16a3*) and the importer MCT1 (*Slc16a1*), also known as the lactate shuttle. To determine whether lactate produced in Sertoli cells could be transported into the germ cells through this shuttle, we examined the presence of MCT4 and MCT1 proteins by immunofluorescence and found that the lactate exporter (MCT4) was predominantly present in SOX9-positive Sertoli cells, whereas the importer (MCT1) was found in germ cells (Figure 4C). These data indicate that the machinery to transport lactate from Sertoli to germ cells is in place and support the notion of metabolic coupling between these cell populations during fetal development.

To investigate whether this metabolic coupling is required for germ cell survival, we injected a dual MCT1 and MCT4 inhibitor (syrosingopine)^32^ or vehicle solution into pregnant female mice at the onset of embryonic sex determination (E10.5 and E11.5) and examined the testes from the XY embryos at the height of glycogen deposition (E12.5) (Figure 4D). Syrosingopine treatment *in vivo* did not affect Sertoli cell fate; however, the number of HuC/D+ germ cells^33^ significantly decreased (Figures 4D and 4E). This result phenocopied the glycogen depletion results (Figure 3), consistent with a model in which glycogen is metabolized into lactate in Sertoli cells and then transported to germ cells through the lactate shuttle during this pivotal window of testis differentiation.

### The decreased germ cell numbers due to inhibition of glycogen breakdown cannot be rescued by providing exogenous lactate or glucose

Based on the result that inhibition of glycogen degradation resulted in lower levels of lactate (Figures 4A and 4B), we wondered if exogenous lactate could rescue germ cell loss in GPI-treated gonads. To test this hypothesis, we cultured embryonic testes with either GPI (75 μM) or GPI (75 μM) plus sodium lactate (25 mM) (Figures S4A and S4B). Consistent with our previous results, treatment with GPI alone led to a reduction of germ cell number (Figure S4E). Contrary to our expectations, supplementation of GPI-treated gonads with an exogenous supply of lactate (25 mM sodium lactate) failed to rescue the germ cell phenotype (Figures S4A, S4B, and S4E), suggesting that lactate cannot fully compensate for the inhibition of glycogen breakdown. One possible explanation is that germ cells, which are closely surrounded by Sertoli cells and enclosed in the center of testis cords, do not have direct access to extracellular lactate. Because Sertoli cells do not express the lactate importer, they cannot act as intermediaries in its uptake and distribution. To evaluate if glucose could be used as a source for lactate generation in Sertoli cells, bypassing glycogen degradation, we cultured the embryonic testes with either GPI or GPI with 25 mM of D-glucose (Figures S4C-S4E). Despite a small, but non-significant, increase in germ cell number (Figure S4D), glucose supplementation, similar to lactate supplementation, failed to lead to normal germ cell numbers (Figures S4C-S4E).

Overall, these findings suggest that Sertoli cells accumulate glycogen and break it down within a transient developmental window to support germ cell survival, as neither Sertoli cells nor germ cells can rely on external sources of carbons, such as glucose and lactate, during this period. This reveals a metabolic pairing during a sensitive period of testicular development that could have long-term effects on male fertility.^34-36^

## DISCUSSION

As glycogen deposition is one of the earliest recognized sexual dimorphisms during mouse gonadal sex differentiation,^25^ it has been an open question whether glycogen directly impacts the differentiation process of the male gonad. Here, we found that neither glycogen synthesis nor its breakdown is required for SOX9 expression, Sertoli cell differentiation, or testicular cord formation. This was unexpected, given previous findings that high extracellular glucose is essential for SOX9 expression and testicular differentiation,^26^ suggesting that glucose derived from glycogen stores and glucose acquired through uptake may have different metabolic fates during early gonadal development.

Disrupting glycogen metabolism in embryonic Sertoli cells, through an organ culture or genetic approach, significantly reduced the number of testicular germ cells. We investigated whether this phenotype could be explained by loss of lactate, a common product of glycogen breakdown and a known nutrient for adult spermatogonia.^14,31^ We found that levels of lactate are reduced when either deposition or breakdown of glycogen is blocked. Furthermore, blocking lactate intercellular transport *in vivo* phenocopies the germ cell loss phenotype we observed when glycogen was depleted. These findings support the model that lactate derived from compartmentalized glycogen in Sertoli cells is an essential metabolite for germ cell survival.

Matoba et al. identified and characterized the sex-specific glycogen accumulation in the developing testis using PAS staining.^25^ A PAS+ signal was first detected between E11 and E11.5 (14–18 ts), coinciding with the expression of SOX9 in Sertoli cells.^25^ Interestingly, when we used a glycogen-specific antibody,^27^ we were unable to detect a glycogen signal in SOX9+ E11.5 testis, despite robust staining in the notochord. These differences could be attributed to different factors, including strain-specific developmental timing (outbred ICR vs. inbred C57BL/6)^37^ or differences in sensitivity and specificity between the two glycogen detection methods. While PAS staining lacks specificity, detecting a range of polysaccharides, including glycogen, it can identify glycogen precursors such as unbranched glucose chains, which may not be recognized by the antibody. Consequently, while PAS staining is likely more sensitive for detecting early or precursor forms of glycogen, antibodybased detection offers greater specificity for mature glycogen, highlighting the value of combining both approaches to capture the dynamics of glycogen accumulation during early testicular development.

Two orthogonal methods, the *in vitro* chemical inhibition of glycogen degradation (GPI inhibitor) and the *in vivo* cKO of *Ppp1r3c* (an enzyme required for glycogen deposition), were used here to interrogate the role of glycogen during sex determination. Although these two approaches examined the same questions from different angles, together they provide a broader understanding of the function of stored glycogen during testis differentiation. For example, stopping glycogen breakdown may trap essential glycolytic metabolites while allowing normal function of glycogen branching machinery. Conversely, stopping glycogen buildup impairs normal glycogen branching machinery but does not lock limited glucose into glycogen deposits. By combining these two methods, we can be more confident that the phenotypes are not secondary to the reduced systemic metabolic input or altered metabolic signaling.

Metabolic coupling similar to that which we characterized between fetal Sertoli and germ cells is also observed in other systems. In the central nervous system, astrocytes, which are essential for the blood-brain barrier formation and maintenance, take up glucose from capillaries to replenish their glycogen storage, which can be rapidly turned over to lactate and exported through the lactate shuttle when neurons require energy.^31,38-41^ Similarly, postnatal Sertoli cells establish the blood-testis barrier and appear to stockpile glycogen and convert it to lactate as a source of energy for germ cells.^42-48^ However, a role for metabolic coupling between Sertoli cells and early germ cells in the fetal testis had not been explored previously. We discovered that the metabolic interplay between supporting cells and germ cells occurs as soon as germ cells arrive at the fetal testis. Interestingly, this developmental period coincides with the differentiation window when male germ cells switch from glycolytic metabolism to oxidative phosphorylation.^49,50^ It is likely that lactate derived from glycogen degradation in Sertoli cells could act as a key enabler of this switch *in vivo*, as lactate allows an expedited metabolic path to oxidative phosphorylation without the energetic requirements of glycolysis. Directly testing this hypothesis will be necessary to determine the full mechanism of Sertoli cell-directed male germ cell metabolic reprogramming.

It is unclear if germ cell recovery is due to the successful completion of a metabolic switch in germ cells or the enhanced availability of nutrients once Sertoli cells have completed the high-energy demands of cord formation. Moreover, although we detect a recovery of germ cell number when glycogen synthesis is perturbed, our current data do not allow us to determine whether these remaining cells are developmentally normal and capable of completing spermatogenesis and generating functional gametes. Due to the lack of an appropriate genetic model system, we were unable to assess whether germ cell loss caused by lactate transport inhibition also recovers over time. Future research is necessary to resolve these limitations. Recent evidence in *Drosophila melanogaster* parallels our findings in the fetal mouse, showing that adult Sertoli cells shuttle lactate to germ cells to support their survival.^51^ The fact that such a mechanism is observed in two evolutionarily distant species suggests that lactate-mediated metabolic coupling between support cells and the germline is a conserved requirement for fertility.

Chemical inhibition of glycogen degradation led to a significant reduction in available intracellular lactate, impairing the ability of cells to meet germ cell demands. Given this deficit, it was expected that exogenous supplementation with glucose (product of glycogen degradation) or lactate would bypass the need for glycogen-derived energy.^31,52^ Surprisingly, supplementation with either lactate or glucose failed to rescue the germ loss phenotype. These findings suggest that glycogen accumulation is essential to support a critical developmental window during which neither Sertoli nor germ cells can rely on external carbon sources. Notably, the developing testis undergoes major structural changes during the period of active glycogen metabolism (E11.5–E13.5),^19^ including an extensive vascular remodeling and testis cord formation.^53,54^ The morphological reorganization of the XY gonad likely imposes high energy demands, prompting Sertoli cells to use compensatory mechanisms, such as glycogen storage and compartmentalization, to simultaneously support the metabolic needs of developing male germ cells. Male germ cells clonally proliferate, differentiate, and die during this temporally constrained period, yielding a selected cohort that ensures fertility in adulthood.^34,35^ It is unclear whether metabolic deficits or the delay in germ cell incorporation into the gonad during this developmental period disrupts the timing of germ cell differentiation. It has been suggested that disruptions in germ cell differentiation result in lasting impacts on stemness and fertility. Abnormal germ cells are thought to serve as precursors for most testicular cancers,^55^ and accumulating evidence suggests that these tumors exhibit distinct metabolic features, including enhanced glycolytic activity.^56-58^ Future studies will be needed to determine whether metabolic perturbations in germ cells contribute to tumorigenesis.

Together, these results provide a compelling example of the emerging field of metabolofertility,^59^ highlighting a critical window of metabolic sensitivity during which male germ cells depend on lactate derived from Sertoli cell glycogen stores to populate the newly forming testis cords. This work adds to a growing body of evidence linking metabolism and fertility, demonstrating how metabolic status, pathways, and intercellular metabolite exchange influence reproductive function across multiple levels, including gametogenesis.

### Limitations of the study

While we observe reduced lactate production upon perturbation of glycogen metabolism and show that lactate transport is required for germ cell maintenance, we are unable to directly determine whether lactate derived specifically from glycogen metabolism is required for germ cell survival. Moreover, although germ cell numbers recover following disruption of glycogen synthesis, our data do not address whether these cells are developmentally normal or capable of completing spermatogenesis. In addition, due to the lack of an appropriate genetic model, we could not assess whether germ cell loss caused by inhibition of lactate transport recovers over time. Similarly, the use of a pharmacological inhibitor to target *ex vivo* all three PYG enzymes may also affect non-Sertoli cell types. Future studies using cell-specific genetic approaches will be required to address these limitations.

## STAR★METHODS

### EXPERIMENTAL MODEL AND STUDY PARTICIPANT DETAILS

#### Animals

C57BL/6J mice were purchased from Jackson Laboratory (stock number 000664). *Nr5a1-Cre* mice (B6D2-g(Nr5a1-cre)2Klp) were generated by Keith Parker and maintained on a C57BL/6 background.^61^ *Ppp1r3c* floxed mice^29^ were obtained from the Saltiel Lab at UCSD and maintained on a C57BL/6 background.

Pairs were set up overnight and females were screened for the presence of vaginal plug the next morning (E0.5). Embryos were staged by tail somite number upon embryonic harvest. For genotyping, DNA was isolated from individual embryos by overnight tissue dissociation in proteinase K, followed by 70% isopropyl alcohol precipitation. Genotyping was performed by PCR and subsequent agarose gel electrophoresis. (*Ppp1r3c*: CCTTTATAGTTGGACCTGTCATGG, TCTACAGTCTAGCTCTGTGCTTGG, expected band size: 656bp wildtype *Ppp1r3c*, 741bp floxed *Ppp1r3c*; *Nr5a1-Cre*: GAACCTGATGGACATGTTCAGG, AGTGCGTT CGAACGCTAGAGCCTGT, expected band size: 320bp; Sex genotyping: TCATGTCCATCAGGTGATGG, CAATGTGGACCATGA CATTG, ATGGACACAGACATTGATGG, expected band size: 420bp *utx*, 288 bp *uty*).

All animal procedures at the National Institute of Environmental Health Sciences were approved by NIEHS Animal Care and Use Committee and are in compliance with a NIEHS-approved animal study proposal (2010-0016). At Duke University Medical Center, all mice were housed in accordance with National Institutes of Health guidelines and the approval of the Duke University Medical Center Institutional Animal Care and Use Committee (A089-20-04 9N).

### METHOD DETAILS

#### GPI gonadal cultures

Individual gonadal cultures were performed using a modified version of the agar gonad culture method.^62^ Agar slabs suitable for 48-well plates were generated using newly designed 3D-printed molds (Figure S4F). Briefly, 150 mg of BactoAgar (214010, Difco Labs) was dissolved in 10 mL of complete media, consisting of 10% heat-inactivated fetal bovine serum (16140071, Gibco), 1X Penicillin-Streptomycin (P0781, Sigma-Aldrich) in 1X phenol red-free DMEM/F12 (1:1) (21041-025, Gibco). The solution was microwaved in 5 s intervals until fully dissolved. Then, 90 μL of the agar solution was added to each mold and solidified agar slabs were transferred to a 48 well plate.

Individual paired gonad-mesonephros complexes were isolated from E12.5 C57BL/6 mouse embryos in cold PBS. Gonads were transferred to the agar slabs containing 180 μL of complete media supplemented with either CP 316819 inhibitor (GPI) (PZ0189, Sigma-Aldrich) at the desired concentration or vehicle solution (DMSO). Gonads were cultured for 48 h at 37°C in 5% CO2, replacing 100 μL of fresh media with an inhibitor or vehicle every 24 h. Cultured gonad-mesonephros complexes were imaged in a Leica MZ16 Stereo Microscope with a Leica MC190 HD Microscope Camera. For rescue experiments, media was supplemented with 25 mM of Sodium L-Lactate (L7022, Sigma-Aldrich) or 25 mM of D-glucose (4912-12, Macron Chemicals).

#### Immunofluorescence and histological analyses

C57BL/6 fetal gonad–mesonephros complexes were fixed in 4% PFA overnight at 4°C. Samples were then washed in PBS, stored at 4°C in 70% ethanol and paraffin embedded. 5 μm paraffin sections were dewaxed and rehydrated. Cultured gonads were collected and fixed in 4% PFA overnight at 4°C. Samples were washed in PBS and cryo-protected in 30% sucrose, blocked in OCT and snap frozen. 10 μm frozen sections were cut. Paraffin sections and cryosections were subjected to citrate-based antigen retrieval (Vector Labs, H-3300-250). Samples were transferred to the Sequenza manual immunohistochemistry system, blocked and permeabilized in 0.1% Triton X-100 in 1X PBS with 5% normal donkey serum for 1h at RT. Slides were incubated with primary antibodies diluted in blocking buffer at 4°C overnight. Samples were then washed and incubated with secondary antibodies diluted in blocking buffer at room temperature for 1h. Slides were washed and autofluorescence was quenched using the TrueView Autofluorescence Quenching Kit (Vector Labs, SP-8400). Samples were counterstained with DAPI (Invitrogen, D1306) and mounted using ProLong Diamond Antifade Mountant (Invitrogen, P36970). Imaging was performed on a Zeiss LSM 900 confocal microscope using Zen software. Brightness and contrast of images were adjusted using FIJI.^60^

For *Ppp1r3c* mice, gonad–mesonephros complexes were dissected in PBS and fixed in 4% PFA for 30 min rocking at room temperature, then dehydrated in an increasing methanol series for long-term storage at −20°C. Samples for cryo-sectioning were gradually rehydrated, cryoprotected through a sucrose gradient (10%, 15%, 20%, and 30%), embedded in OCT and moved to −80°C for a minimum of 12 h before cryo-sectioning. Blocks were serially sectioned at 10–16 μm and placed on slides that were stored at −20°C until ready to use. For immunostaining, sections were rehydrated in PBS, permeabilized in PBST (0.1% Triton X-100), and blocked for 1 h at room temperature (PBS 0.1% Triton X-100, 3% BSA, 10% horse serum). Sections were incubated in primary antibodies diluted in blocking solution at 4°C overnight. Following three 10-min washes in PBS 0.1% Triton X-100, sections were incubated for 2 h at room temperature with DAPI and secondary antibodies diluted in blocking solution. Following three 10-min washes in PBST, slides were mounted for imaging with polyvinyl alcohol mounting solution and stored at 4°C. Sections were imaged using the Andor Dragonfly Spinning Disk confocal microscope.

#### Periodic acid schiff (PAS) staining

The PAS staining protocol was adapted for use on cryo-sectioned tissue. Briefly 10–16 μm sections were transferred to superfrost PLUS slides and stored at −20°C until use. Using a pap pen to contain liquid, a 0.5% Periodic acid solution was added to each slide for 5 min at room temperature. Each slide was rinsed 3 times in dH_2_0 and Schiff’s solution was added and incubated for 15 min at room temperature. Each slide was rinsed 3 times in lukewarm tap water and dehydrated in a 25%, 65%, and 100% ethanol gradient. All sectioned samples were mounted in Polyvinyl Alcohol mounting media using #1.5 coverslips and sealed with nail polish. Stained sections were brightfield imaged using an Zeiss axio imager upright microscope.

#### Whole-mount immunofluorescence

Cultured gonads were collected, fixed in 4:1 methanol:DMSO and stored at −20°C for at least 24hs. Samples were washed with 50% methanol diluted in 1X PBS for 30 min at room temperature, followed by 3 one-hour washes with blocking solution (10% donkey serum and 1% Triton in 1X PBS) at room temperature. Samples were incubated overnight at 4°C in blocking solution containing primary antibodies. After primary incubation, samples were washed 3 times with blocking buffer for 1 h each at 4°C and incubated with secondary antibodies in blocking solution overnight at 4°C. Samples were dehydrated by 1-h washes in increasing methanol concentrations (25%, 50%, 75% with DAPI, and 100%). Tissues were cleared with 1:2 benzyl alcohol:benzyl benzoate (BABB) for at least 24 h and imaged on a Zeiss LSM 900 confocal microscope using Zen software.

For *Ppp1r3c* mice, gonad–mesonephros complexes were dissected in PBS and fixed in 4% PFA for 30 min rocking at room temperature, then taken up a methanol gradient to 100% MeOH for long-term storage at −20°C. After gradual rehydration into PBS, tissues were permeabilized in PBS 0.1 to 0.5% Triton X-100 for 1 h at room temperature and transferred into blocking solution (PBS 0.1 to 1% Triton X-100, 3% BSA, 10% horse serum) for 2 h. Samples were incubated with primary antibodies diluted in blocking solution overnight at 4°C. Following three 30-min washes in PBS 0.1 to 1% Triton X-100, tissues were incubated with secondary antibodies and DAPI diluted in blocking solution overnight at 4°C. Tissues were washed three times in PBS/Triton X-100 for 1 h at room temperature, mounted for imaging in 2% Agarose in Cubic R2 clearing media^63^ and stored at 4°C until imaging on the Andor Dragonfly Spinning Disk confocal microscope. For gonad samples ranging from E11.5-E12.5, the concentration of Triton X-100 was 0.5%, while for samples from E13.5 and above, the concentration was increased to 1%.

#### RNA extraction

Vehicle and GPI treated gonads were manually dissected from the mesonephros and RNA was extracted from each vehicle and GPI treated gonadal pair for a total of 5 paired samples. Two E12.5 gonadal pairs (control or Ppp1r3c cKO) were pooled per sample, for a total of 3 samples per group. RNA was extracted using the Arcturus PicoPure RNA Isolation Kit (Applied Biosystems, KIT0204). Samples were dissociated in 100 μL of extraction buffer with a microtube homogenizer and incubated at 42°C for 30 min, then frozen at −80°C for at least one hour. RNA was precipitated with 100 μL of 70% ethanol and loaded into a pre-conditioned column. Samples were subjected to DNA removal using the RNase-Free DNase Set (Qiagen, #79254). Samples were eluted in 12 μL of elution buffer. RNA was quantified using Qubit RNA High Sensitivity kit (Q32852, ThermoFisher Scientific). RNA quality was assessed using High Sensitivity RNA or RNA ScreenTape assay, in a TapeStation (Agilent).

#### RNA sequencing

For paired vehicle and GPI treated samples, libraries were made using Tecan’s Ovation RNA-Seq System V2 followed by Tecan’s Celero EZ DNA-Seq. For control or Ppp1r3c cKO samples, 250 ng of RNA were used for libraries preparation using the TruSeq Stranded mRNA Library Prep protocol (Illumina, #20020594). Libraries were prepared and sequenced by the NIEHS Epigenomics and DNA Sequencing Core as paired-end 151-mers on an Illumina NextSeq 500 instrument.

#### RNA-seq analysis

Read pairs were mapped to the mm10 reference genome via STAR v2.5.1b^64^ with parameters“–outSAMattrIHstart 0 –outFilterType BySJout –alignSJoverhangMin 8 –limitBAMsortRAM 55000000000 –outSAMstrandField intronMotif –outFilterIntronMotifs RemoveNoncanonical”. Counts per gene were determined via featureCounts (Subread v1.5.0-p1)^65^ with parameters“-s2 -Sfr -p” for the Ppp1r3c dataset (reverse-stranded libraries) or with parameters“-s0 -Sfr -p” for the GPI dataset (unstranded libraries). Evaluated gene models were taken from the NCBI RefSeq Curated annotations as downloaded from the UCSC Table Browser on April 21 2021. DESeq2 v1.42.0^66^ was used for principal component analysis and identification of differentially expressed genes (threshold set at FDR 0.05); for the GPI dataset, the individual mouse identifier is included as a variable in the design formula to account for sample pairing. Gene ontology and pathway analysis were analyzed using Enrichr^67-69^ and graphed using R studio.

#### Single-cell RNA-seq data mining

Dataset for this analysis was taken from Chen et al.,^28^ with Dot Plot generated as part of the ShinyCell package (v. 2.1.0).^70^ Differentially expressed genes (DEG) between male and female somatic clusters were determined using the“FindMarkers” function in Seurat (v. 4.3.0)^71^ based on the Wilcoxon Rank-Sum test with adjusted *p*-value <0.05.

#### Lactate measurement

Lactate concentration was measured using the Lactate-Glo Assay (J5021, Promega). Briefly, single cultured gonads or E12.5 *Ppp1r3c* control or cKO gonadal pairs were dissected from the mesonephros, snap-frozen on dry ice and stored at −80°C until processing. For sample preparation, each sample was homogenized in 50 μL of homogenization buffer (50mM Tris, pH 7.5) and 6.25 μL of inactivation solution (0.6N HCl) using a microtube homogenizer. Immediately after homogenization, 6.25 μL of neutralization solution (1M Tris base Trizma) was added. Protein concentration was determined using 10 μL of the sample with the Qubit Protein Assay (Q33211, ThermoFisher Scientific). For lactate detection, 50 μL of the processed sample was mixed with 50 μL of freshly prepared lactate detection reagent and incubated at room temperature for 60 min. A standard curve was generated using serial dilution of lactate, starting at 200 μM, to interpolate lactate concentration from the samples. Luminescence was recorded using the Promega GloMax Discoverer microplate reader (GM3000, Promega). Lactate concentration per sample was determined by interpolation in the standard curve and normalized to protein content. Data visualization and statistical analysis (two-tailed *t* test) was performed using GraphPad Prism.

#### Syrosingopine treatment *in vivo*

Five milligrams of Syrosingopine (medchemexpress, CAS: 84-36-6, CAT: HY-N4115) dissolved in corn oil was injected intraperitoneally into C57BL/6 pregnant mothers once on E10.5 and again on E11.5. Control mice were injected with vehicle (DMSO in corn oil) at the same time points. E12.5 gonad-mesonephros complexes were collected and processed for whole mount immunofluorescence.

### QUANTIFICATION AND STATISTICAL ANALYSIS

#### PPP1R3C MFI quantification

PPP1R3C mean fluorescence intensity (MFI) was quantified from E11.5, E12.5, and E13.5 male and female gonadal sections using FIJI. The gonadal area, excluding the surface epithelium, was defined as the region of interest (ROI), and the mean fluorescence intensity was measured for each sample. For each developmental stage, values were normalized to the corresponding female mean. Data visualization and statistical analysis (multiple two-tailed t-tests) were performed in GraphPad Prism.

#### Germ cell number quantification

Germ cell number in cultured gonads was quantified using FIJI on whole mount samples stained with DDX4 immunofluorescence.^60^ For *Ppp1r3c* mice, germ cells were quantified using FIJI and an in house trained CellposeSAM model^72^ by segmenting TRA98 positive cells in subsequent 10 μm maximum intensity projection through each whole mount gonad. For Syrosingopine treated gonads the HuC/D positive germ cells^33^ were counted by hand in FIJI following the same 10um projection paradigm. Data visualization and statistical analysis (two-tailed *t* test) and one-way ANOVA was performed using GraphPad Prism.

## Supplementary Material

1

2

3

4

Supplemental information can be found online at https://doi.org/10.1016/j.celrep.2026.117069.

## Figures and Tables

**Table:** KEY RESOURCES TABLE

REAGENT or RESOURCE	SOURCE	IDENTIFIER
Antibodies		
Glycogen	Otto Baba^27^	IV58B6
AMH	Santa Cruz	Cat#sc-6886; RRID: AB_649207
FOXL2	Abcam	Cat# ab5096; RRID: AB_304750
FOXL2	Dagmar Williams	RRID: AB_2687958
NR5A1	Cosmo Bio	Cat# KO610; RRID: AB_3696859
PPP1R3C	Proteintech	Cat# 29424-1-AP; RRID: AB_2918304
PYGB	Proteintech	Cat# 12075-1-AP; RRID: AB_2174885
SOX9	Milipore	Cat# AB5535; RRID: AB_2239761
SOX9	R&D systems	Cat# AF3075; RRID: AB_2194160
SRY	Dagmar Williams	RRID: AB_2687957
Ki-67	BD	Cat# 550609; RRID: AB_393778
CC3	Cell Signaling	Cat# 9661; RRID: AB_2341188
DDX4	Abcam	Cat# ab13840; RRID: AB_443012
TRA98	Abcam	Cat# ab82527; RRID: AB_1659152
MCT1	Milipore	Cat# AB1286; RRID: AB_11212410
MCT4	Santa Cruz	Cat# sc-376140; RRID: AB_10992036
HuC/D	Vanda Lennon	RRID: AB_2813895
Alexa Fluor 488-Anti-Mouse	Invitrogen	Cat# A21202; RRID: AB_141607
Alexa Fluor 488-Anti-Rabbit	Invitrogen	Cat# A21206; RRID: AB_2535792
Alexa Fluor 488-Anti-Rat	Invitrogen	Cat# A21208; RRID: AB_2535794
Alexa Fluor 488-Anti-Goat	Invitrogen	Cat# A11055; RRID: AB_2534102
Alexa Fluor 568-Anti-Mouse	Invitrogen	Cat# A10037; RRID: AB_11180865
Alexa Fluor 568-Anti-Rabbit	Invitrogen	Cat# A10042; RRID: AB_2534017
Alexa Fluor 568-Anti-Rat	Invitrogen	Cat# A78946; RRID: AB_2910653
Alexa Fluor 568-Anti-Goat	Invitrogen	Cat# A11057; RRID: AB_142581
Alexa Fluor 647-Anti-Mouse	Invitrogen	Cat# A31571; RRID: AB_162542
Alexa Fluor 647-Anti-Rabbit	Invitrogen	Cat# A31573; RRID: AB_2536183
DyLight650-Anti-Rat	Invitrogen	Cat# SA510029; RRID: AB_2556609
Alexa Fluor 647-Anti-Goat	Invitrogen	Cat# A21447; RRID: AB_2535864
Cy5-Anti-Chicken	Jackson	Cat# 703-175-155; RRID: AB_2340365
Chemicals, peptides, and recombinant proteins		
CP 316819 (GPI)	Sigma-Aldrich	Cat# PZ0189
Syrosingopine	medchemexpress	Cat# HY-N4115
Sodium L-Lactate	Sigma-Aldrich	Cat# L7022
D-glucose	Macron Chemicals	Cat# 4912-12
Critical commercial assays		
Arcturus^™^ PicoPure^™^ RNA Isolation Kit	Applied Biosystems	Cat# KIT0204
RNase-Free DNase Set	Qiagen	Cat# 79254
Qubit^™^ RNA High Sensitivity kit	ThermoFisher Scientific	Cat# Q32852
Tecan’s Ovation^®^ RNA-Seq System V2	Tecan	Cat# 7102
Tecan’s Celero^™^ EZ DNA-Seq	Tecan	Cat# 30188860
TruSeq Stranded mRNA Library Prep	Illumina	Cat# 20020594
Lactate-Glo Assay	Promega	Cat# J5021
Qubit Protein Assay	ThermoFisher Scientific	Cat# Q33211
High Sensitivity RNA ScreenTape + Sample Buffer	Agilent	Cat# 5067–5578/80
RNA ScreenTape assay + Sample Buffer	Agilent	Cat# 5067–5576/77
Deposited data		
*Ppp1r3c* cKO bulk RNA-seq	This paper	GEO: GSE293016
GPI bulk RNA-seq	This paper	GEO: GSE293017
Experimental models: Organisms/strains		
C57BL/6	Jackson Laboratory	000664
B6;D2-Tg(Nr5a1-cre)2Klp/HyJ	Jackson Laboratory	037533
*Ppp1r3c* floxed	Saltiel Laboratory	Keinan et al.^29^
Software and algorithms		
ImageJ/FIJI	Schindelin et al.^60^	RRID:SCR_003070
RStudio	RStudio	RRID:SCR_000432
Prism	GraphPad Software	RRID:SCR_002798

## Highlights

- Fetal Sertoli cells transiently accumulate and consume glycogen
- Glycogen is not essential for Sertoli fate
- Loss of Sertoli cell glycogen affects gonadal lactate levels and germ cell number
- Blocking the lactate shuttle phenocopies loss of glycogen
