# Supplementary material for: Glycogen and lactate metabolism in mouse fetal Sertoli cells sustain the germ line
Source: Cell Rep. Author manuscript; Available in PMC 2026 May 14. (PMC13173509; doi:10.1016/j.celrep.2026.117069)
Supplement: 1 [file NIHMS2160133-supplement-1.pdf]

Cell Reports, Volume *45*

Supplemental information

Glycogen and lactate metabolism in mouse fetal Sertoli cells sustain the germ line

Martín A. Estermann, Joseph Sheheen, Sara A. Grimm, Boris Tezak, Yu-Ying Chen, Tsuyoshi Morita, Humphrey H.-C. Yao, and Blanche Capel

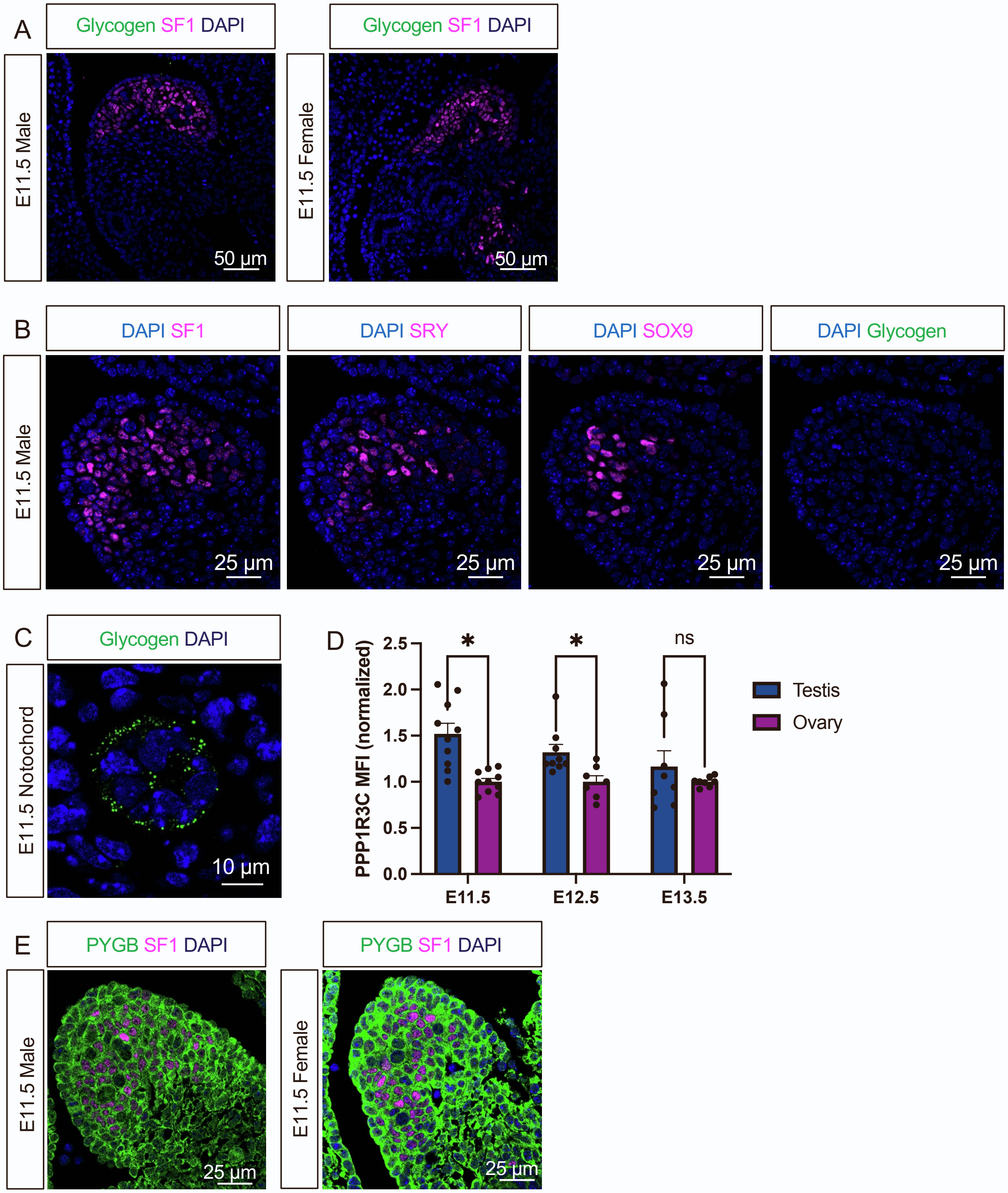
A
Glycogen SF1 DAPI
E11.5 Male
50 µm
Glycogen SF1 DAPI
E11.5 Female
50 µm
B
DAPI SF1
DAPI SRY
DAPI SOX9
DAPI Glycogen
E11.5 Male
25 µm
C
Glycogen DAPI
E11.5 Notochord
10 µm
D
PPP1R3C MFI (normalized)
2.5
2.0
1.5
1.0
0.5
0.0
*
*
ns
E11.5
E12.5
E13.5
Testis
Ovary
E
PYGB SF1 DAPI
E11.5 Male
25 µm
PYGB SF1 DAPI
E11.5 Female
25 µm

Figure S1: Based on antibody staining, glycogen does not accumulate before E12.5.

(A) Immunofluorescence for glycogen (green) and the supporting cell marker SF1 (magenta) in transverse E11.5 male and female gonadal sections. Samples were counterstained with DAPI (blue).

(B) Immunofluorescence for SF1, SRY or SOX9 (magenta) and glycogen (green) in transverse E11.5 male gonadal sections. Samples were counterstained with DAPI (blue).

(C) Immunofluorescence for glycogen (green) in transverse E11.5 male notochord section, used as an within-experiment control. Samples were counterstained with DAPI (blue).

(D) PPP1R3C mean fluorescence intensity (MFI) in E11.5, E12.5, and E13.5 testes and ovaries. Values are normalized to the corresponding female sample at each developmental stage. Bars represent mean±s.e.m., n≥7. Multiple two-tailed t-test. ns adjpvalue>0.05; * adjpvalue<0.05.

(E) Immunofluorescence for PYGB (green) and supporting cell marker SF1 (magenta) in transverse E11.5 male and female gonadal sections. Samples were counterstained with DAPI (blue).

Figure S2

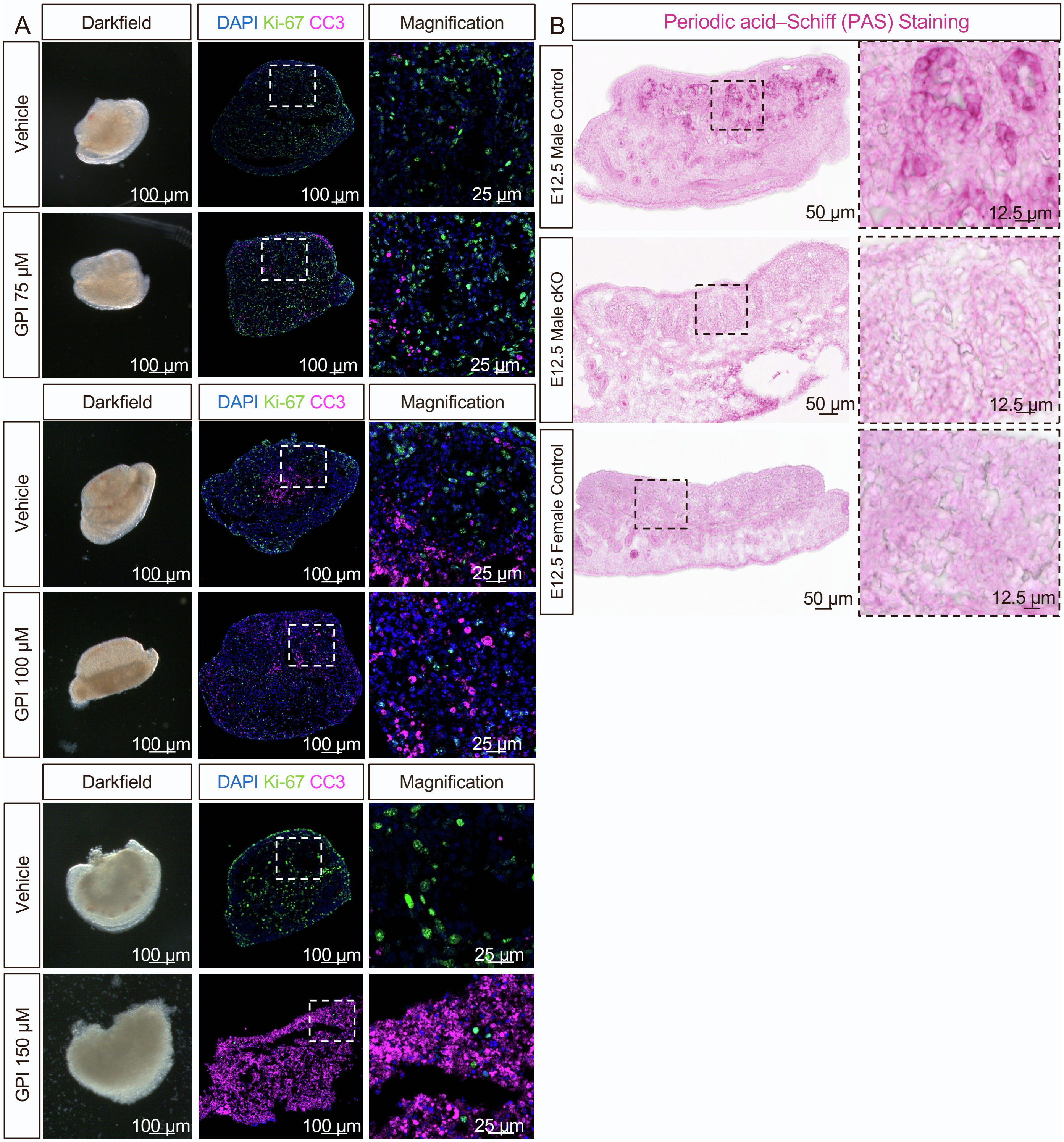
A
Darkfield
DAPI Ki-67 CC3
Magnification
Vehicle
GPI 75 µM
GPI 100 µM
GPI 150 µM
100 µm
25 µm
B
Periodic acid–Schiff (PAS) Staining
E12.5 Male Control
E12.5 Male cKO
E12.5 Female Control
50 µm
12.5 µm

Figure S2: Characterization of glycogen metabolism perturbation models.

(A) Dose response of gonads treated with GPI at different concentrations (75 µM, 100 µM and 150 µM) or their respective vehicle solution (DMSO). Darkfield visualization (left) and immunostaining (right) for proliferation (Ki-67, green) and apoptosis (CC3, magenta). Samples were counterstained with DAPI (blue). White dashed box indicates the magnified area.

(B) PAS staining showing lack of glycogen deposition in E12.5 *Ppp1r3c* cKO male gonads. E12.5 control male and female gonads shown for comparison. Black dashed box indicates the magnified area.

Figure S3

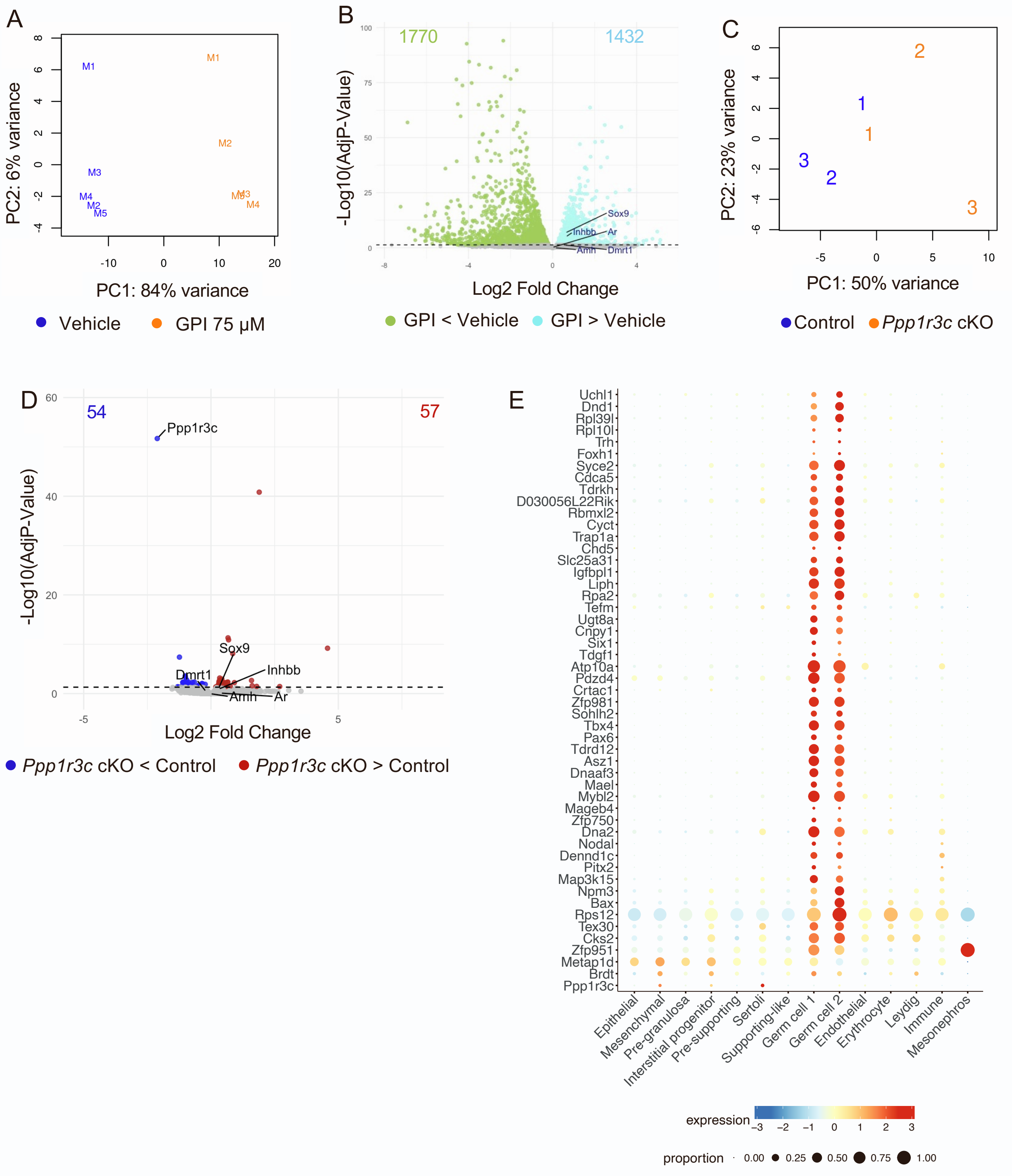
A
PC2: 6% variance
PC1: 84% variance
Vehicle
GPI 75 µM
B
1770
1432
-Log10(AdjP-Value)
Log2 Fold Change
Sox9
Inhbb
Ar
Amh
Dmrt1
GPI < Vehicle
GPI > Vehicle
C
PC2: 23% variance
PC1: 50% variance
Control
Ppp1r3c cKO
D
54
57
Ppp1r3c
Sox9
Inhbb
Dmrt1
Amh
Ar
-Log10(AdjP-Value)
Log2 Fold Change
Ppp1r3c cKO < Control
Ppp1r3c cKO > Control
E
Uchl1
Dnd1
Rpl39l
Rpl10l
Trh
Foxh1
Syce2
Cdca5
Tdrkh
D030056L22Rik
Rbmxl2
Cyct
Trap1a
Chd5
Slc25a31
Igfbpl1
Liph
Rpa2
Tefm
Ugt8a
Cnpy1
Six1
Tdgf1
Atp10a
Pdzd4
Crtac1
Zfp981
Sohlh2
Tbx4
Pax6
Tdrd12
Asz1
Dnaaf3
Mael
Mybl2
Mageb4
Zfp750
Dna2
Nodal
Dennd1c
Pitx2
Map3k15
Npm3
Bax
Rps12
Tex30
Cks2
Zfp951
Metap1d
Brdt
Ppp1r3c
Epithelial
Mesenchymal
Pre-granulosa
Interstitial progenitor
Pre-supporting
Sertoli
Supporting-like
Germ cell 1
Germ cell 2
Endothelial
Erythrocyte
Leydig
Immune
Mesonephros
expression
proportion
0.00
0.25
0.50
0.75
1.00

Figure S3: Transcriptomic characterization of glycogen metabolism perturbation models.

(A) Principal component analysis (PCA) plot of the bulk RNA-seq of paired testes cultured with 75 μM GPI or vehicle solution.

(B) Volcano plot of differentially expressed genes between 75 μM GPI or vehicle treated testes. Green and cyan dots represent downregulated and upregulated genes, respectively, in the GPI treated testes compared with vehicle controls.

(C) Principal component analysis (PCA) plot of the bulk RNA-seq of control and *Ppp1r3c* cKO testes.

(D) Volcano plot of differentially expressed genes between the *Ppp1r3c* cKO and the control testes. Blue and red dots represent downregulated and upregulated genes, respectively, in the cKO testis in comparison to the control.

(E) Dot plot showing the expression pattern of the 54 genes downregulated upon Sertoli cell specific *Ppp1r3c* deletion, mapped onto the major E12.5 gonadal cell populations from the single-nucleus RNA-seq dataset. Dot size represents the proportion of cells within each cell population expressing a given gene, and dot color indicates the average expression level of each gene within the population. The majority of downregulated genes are specifically expressed in germ cells.

Figure S4

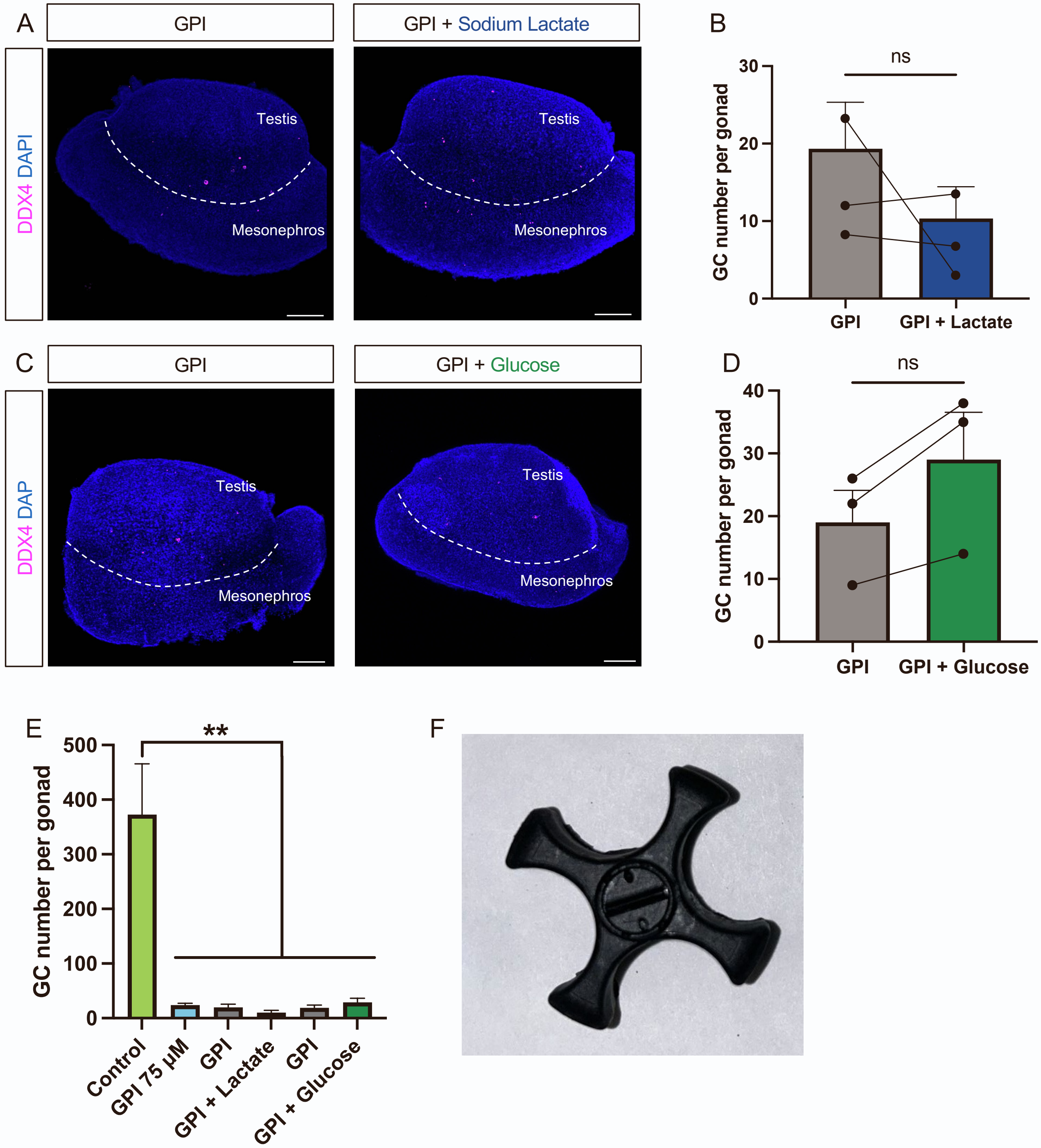
A
GPI
GPI + Sodium Lactate
DDX4 DAPI
Testis
Mesonephros
B
ns
GC number per gonad
30
20
10
0
GPI
GPI + Lactate
C
GPI
GPI + Glucose
DDX4 DAP
Testis
Mesonephros
D
ns
40
30
20
10
0
GC number per gonad
GPI
GPI + Glucose
E
**
500
400
300
200
100
0
GC number per gonad
Control
GPI 75 μM
GPI
GPI + Lactate
GPI
GPI + Glucose
F

Figure S4: Exogenous carbon sources fail to rescue germ cell loss induced by GPI treatment.

(A) 3D projection of DDX4 (germ cell, magenta) whole mount immunostaining in cultured male gonads treated with 75 μM GPI or 75 μM GPI + 25mM sodium lactate. Samples were counterstained with DAPI (blue). Dashed white line delineates the testicular region. Scale bars are 100 μm.

(B) Quantification of the total number of germ cells in male gonads cultured with 75 μM GPI or 75 μM GPI + 25mM sodium lactate. Bars represent mean±s.e.m., n=3. Paired two-tailed t-test. ns p>0.05.

(C) 3D projection of DDX4 (germ cell, magenta) whole mount immunostaining in cultured male gonads treated with 75 μM GPI or 75 μM GPI + 25mM D-glucose. Samples were counterstained with DAPI (blue). Dashed white line delineates the testicular region. Scale bars are 100 μm.

(D) Quantification of the total number of germ cells in male gonads cultured with 75 μM GPI or 75 μM GPI + 25mM D-glucose. Bars represent mean±s.e.m., n=3. Paired two-tailed t-test. ns p>0.05.

(E) Quantification of the total number of germ cells in male gonads cultured with vehicle solution, 75 μM GPI, 75 μM GPI + 25mM sodium lactate or 75 μM GPI + 25mM D-glucose. Bars represent mean±s.e.m., n>3. One-way ANOVA. ** p<0.005.

(F) 3D printed mold used to generate the agar slabs for single gonad cultures.
